# Inflammatory Aneurysm of the Common Iliac Artery With Elevated Serum Levels of Immunoglobulin G4 Manifesting After Endovascular Aneurysm Repair: A Case Report

**DOI:** 10.7759/cureus.82964

**Published:** 2025-04-25

**Authors:** Nozomu Ishikawa, Naoto Yamamoto, Naoki Unno, Masaki Sano, Hiroya Takeuchi

**Affiliations:** 1 Department of Vascular Surgery, Hamamatsu Medical Center, Hamamatsu, JPN; 2 Department of Surgery, Hamamatsu University School of Medicine, Hamamatsu, JPN; 3 Division of Vascular Surgery, Hamamatsu University School of Medicine, Hamamatsu, JPN

**Keywords:** endovascular aneurysm repair, immunoglobulin g4-related disease, inflammatory aneurysm, post-implantation syndrome, steroids

## Abstract

Inflammatory aneurysms (IAs) are characterized by the thickening of the arterial wall and fibrosis of periarterial tissues. Immunoglobulin G4 (IgG4)-related disease (IgG4-RD) is related to IAs, and about half of IA cases are considered IgG4-related. Although some cases of inflammation after endovascular aneurysm repair (EVAR) have been reported, its relationship with the serum levels of IgG4 has rarely been discussed. Here, we report on a patient diagnosed with and treated for an IA with elevated serum levels of IgG4 following EVAR. An 83-year-old man presented with a bilateral common iliac artery aneurysm. We observed no inflammatory features in his vital signs, laboratory test results, or computed tomography (CT) images. The patient was diagnosed with a non-inflammatory bilateral common iliac artery aneurysm. EVAR was performed using an infrarenal bifurcated stent graft (diameter, 31 mm; length, 15 cm; GORE EXCLUDER AAA Endoprosthesis (WL Gore & Associates, Inc., Flagstaff, AZ, USA)), with an ipsilateral limb stent graft (diameter, 12 mm; length, 7 cm; GORE EXCLUDER) deployed in the left external iliac artery and a contralateral limb stent graft (diameter, 12 mm; length, 14 cm; GORE EXCLUDER) deployed in the right external iliac artery. The patient was discharged in good condition. However, signs of inflammation were observed approximately one month after EVAR. CT images demonstrated the periarterial thickening of the common iliac arteries, and 18F-fluorodeoxyglucose positron emission tomography-CT revealed increased metabolic activity overlying the thickened periarterial tissue. The serum levels of IgG4 and soluble interleukin-2 receptor (sIL-2R) were elevated. The patient underwent steroid therapy based on the suspicion of IgG4-related IA of the common iliac arteries, resulting in reductions of inflammatory signs and periarterial thickening. However, when steroids were reduced, hydronephrosis, periarterial thickening, and increased serum IgG4 and sIL-2R levels were observed. The patient was diagnosed with a relapsed IA and treated with an increased steroid dosage. IAs may occur following EVAR. Steroid therapy may be an effective treatment for post-EVAR IAs, similar to common IAs. Long-term follow-up is desirable to monitor patients for the recurrence of inflammation during the treatment of post-EVAR IAs.

## Introduction

Inflammatory aneurysms (IAs) are characterized by the thickening of arterial walls and fibrosis of periarterial tissues [1]. IAs account for 5-10% of arterial aneurysm cases [2]. About half of IA cases are considered immunoglobulin G4 (IgG4)-related [2]. Specifically, Kasashima et al. reported that IgG4-related IA cases accounted for 5% of all surgically treated abdominal aortic aneurysms (AAAs) and 57% of IA cases [3]. Therefore, it is estimated that approximately 1-5% of all AAAs may be IgG4-related. IgG4-related IAs are mostly observed in the abdominal aorta and/or common iliac arteries [4]. Steroid therapy is effective for IAs, and open surgical or endovascular repairs are indicated for large IAs [2]. Here, we describe a case of an inflammatory common iliac aneurysm with elevated serum levels of IgG4 after endovascular aneurysm repair (EVAR).

## Case presentation

**Figure 1:**
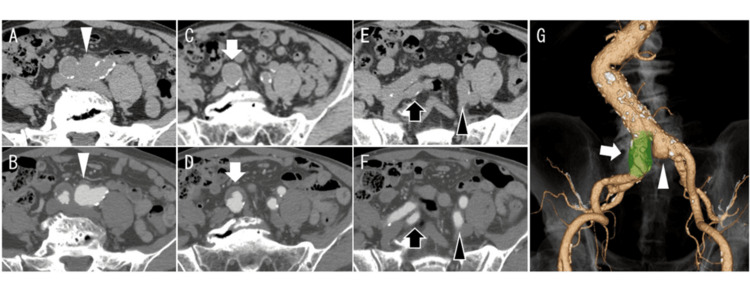
Preoperative CT Panels A, C, and E show unenhanced (plain) CT images, while panels B, D, and F show contrast-enhanced CT images. (A and B) Saccular left common iliac artery aneurysm with a maximum diameter of 28 mm (white arrowhead). (C and D) Right common iliac artery aneurysm with a maximum diameter of 24 mm (white arrow). (E and F) No aneurysmal enlargement is observed in the bilateral internal iliac arteries (black arrow: right side; black arrowhead: left side). (G) Three-dimensional reconstruction image showing the left saccular and right fusiform common iliac artery aneurysms (white arrowhead and white arrow, respectively). CT: computed tomography

An 83-year-old man with a medical history of chronic obstructive pulmonary disease (COPD) presented with a left saccular common iliac artery aneurysm measuring 28 mm in diameter (Figure 1A, 1B) and a right fusiform common iliac artery aneurysm measuring 24 mm in diameter (Figure 1C, 1D), both of which were detected on computed tomography (CT). No abnormal features were observed in the abdominal, thoracic, external, and internal iliac arteries (Figure 1E, 1F). Additionally, there was no evidence of periarterial inflammation or hydronephrosis. The patient was asymptomatic, and his vital signs were normal. Laboratory test results were unremarkable, with no indications of inflammation (white blood cells (WBCs), 6700 cells/μL; C-reactive protein (CRP), 0.8 mg/dL) and no evidence of severe renal dysfunction (serum creatinine, 1.00 mg/dL). The patient was diagnosed with a non-inflammatory bilateral common iliac artery aneurysm (Figure 1G). The left common iliac artery aneurysm was saccular and exhibited rapid growth (6 mm over six months). Due to his advanced age (83 years) and poor general condition with COPD, we planned to perform EVAR.

The use of iliac branch devices may be desirable for maintaining blood flow to the internal iliac arteries and pelvic blood circulation. However, preoperative examination revealed that the patient's vascular anatomy was outside the instructions for using such devices. Thus, we performed three-stage surgery: internal iliac artery embolization performed one side at a time, followed by EVAR from the abdominal aorta to the external iliac arteries.

**Figure 2:**
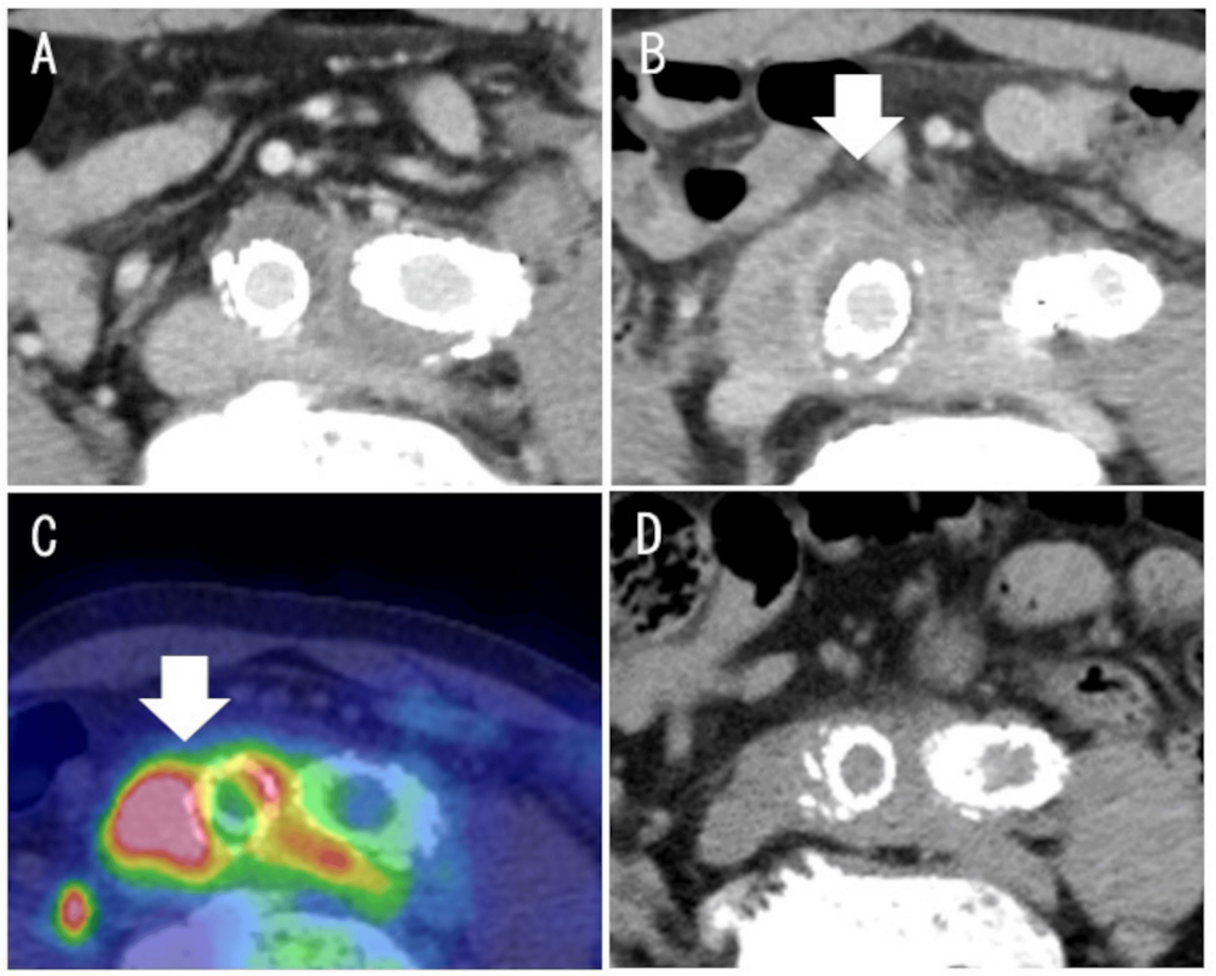
Chronological change in CT images after endovascular aneurysm repair Periarterial thickening was not observed on CT on postoperative day 5 (A); however, it was observed at postoperative month 2 (B), along with steroid reduction. (C) 18F-fluorodeoxyglucose positron emission tomography-CT performed at postoperative month 2 demonstrated increased metabolic activity corresponding to the area of periarterial thickening, with a standardized uptake value of 11.56. (D) Periarterial thickening decreased with steroid therapy at postoperative month 4. White arrows indicate periarterial thickening. CT: computed tomography

After obtaining bilateral femoral access, a bifurcated stent graft (diameter, 31 mm; length, 15 cm; GORE EXCLUDER AAA Endoprosthesis, WL Gore & Associates, Inc., Flagstaff, AZ, USA) was deployed in the infrarenal abdominal aorta. A limb stent graft (diameter, 12 mm; length, 7 cm; GORE EXCLUDER AAA Endoprosthesis) was deployed in the left external iliac artery, and another limb stent graft (diameter, 12 mm; length, 14 cm; GORE EXCLUDER AAA Endoprosthesis) was deployed in the right external iliac artery. Contrast-enhanced CT on postoperative day 5 revealed no endoleaks or abnormal periarterial characteristics (Figure 2A), and the patient was discharged. However, on postoperative day 12, the patient presented with fever, and laboratory tests revealed inflammation (WBCs, 11,700 cells/µL; CRP, 20.3 mg/dL). Infectious complications or post-implantation syndrome (PIS) was suspected. Various culture tests revealed a urinary tract infection, and antibiotic treatment was administered. Although various culture results were negative after antibiotic treatment, inflammation did not substantially improve (WBCs, 10,290 cells/µL; CRP, 15.4 mg/dL). Contrast-enhanced CT subsequently revealed the enhanced thickening of the periarterial tissue of the common iliac arteries, with no changes in the diameters of either of the common iliac aneurysms (Figure 2B). Moreover, 18F-fluorodeoxyglucose positron emission tomography-CT (PET-CT) revealed localized increased abnormal metabolic activity overlying the thickened periarterial tissue in the right common iliac aneurysm with a standardized uptake value of 11.56 (Figure 2C). The serum level of IgG4 was elevated to 241 mg/dL (normal range: 4.8-105 mg/dL), and that of soluble interleukin-2 receptor (sIL-2R) was elevated to 1370 U/mL (normal range: 157-474 U/mL). As no pathological findings were obtained, the patient was diagnosed with suspected IgG4-related IA of the common iliac arteries. Steroid therapy was initiated with oral prednisolone at 30 mg/day (approximately 0.5 mg/kg/day), in line with international recommendations for IgG4-related disease (IgG4-RD) [5], after confirming that various cultures were negative. CT at postoperative month 4 revealed a decrease in the thickening of the periarterial tissue of the common iliac arteries (Figure 2D). The serum levels of IgG4 and sIL-2R decreased to within the normal ranges. This clinical course indicated that the IA responded to steroid therapy.

**Figure 3:**
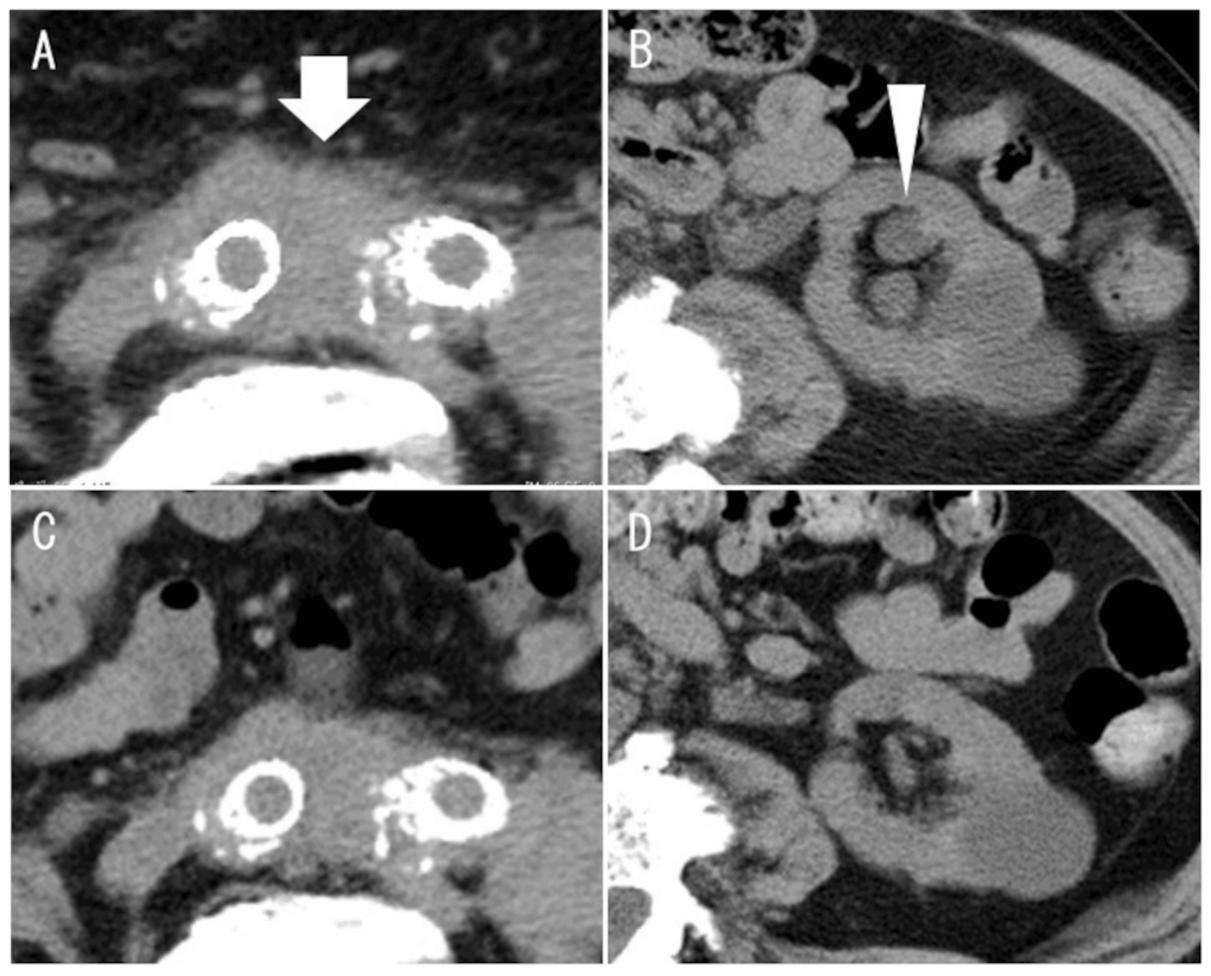
Inflammation recurrence in chronological CT changes CT at postoperative month 12 indicated the recurrence of periarterial thickening (A) (white arrow) and hydronephrosis of the left kidney (B) (white arrowhead), along with steroid reduction. Along with increased steroid dosage, periarterial thickening decreased (C) and hydronephrosis was reduced (D). CT: computed tomography

The prednisolone dose was then gradually reduced to 10 mg/day. One year postoperatively, the patient presented with abdominal pain and was hospitalized. CT images revealed the thickening of the periarterial tissue of the common iliac arteries and grade 1 hydronephrosis on the left side (Figure 3A, 3B) [6]. Hydronephrosis was attributed to ureteral obstruction caused by periarterial tissue thickening. The right ureter remained patent, and serum creatinine levels did not show a significant increase from baseline. Therefore, ureteral stent placement was not required. The serum levels of IgG4 and sIL-2R were elevated to 199 mg/dL and 877 U/mL, respectively. The patient was diagnosed with a relapsed IA in the common iliac artery. Upon relapse, the patient received intravenous methylprednisolone 250 mg/day for three consecutive days, followed by the re-initiation of oral prednisolone at 30 mg/day. The patient's symptoms had decreased, and the serum levels of IgG4 and sIL-2R had decreased to 118 mg/dL and 587 U/mL, respectively. Moreover, CT after two weeks revealed that periarterial thickness and hydronephrosis had decreased (Figure 3C, 3D).

**Figure 4:**
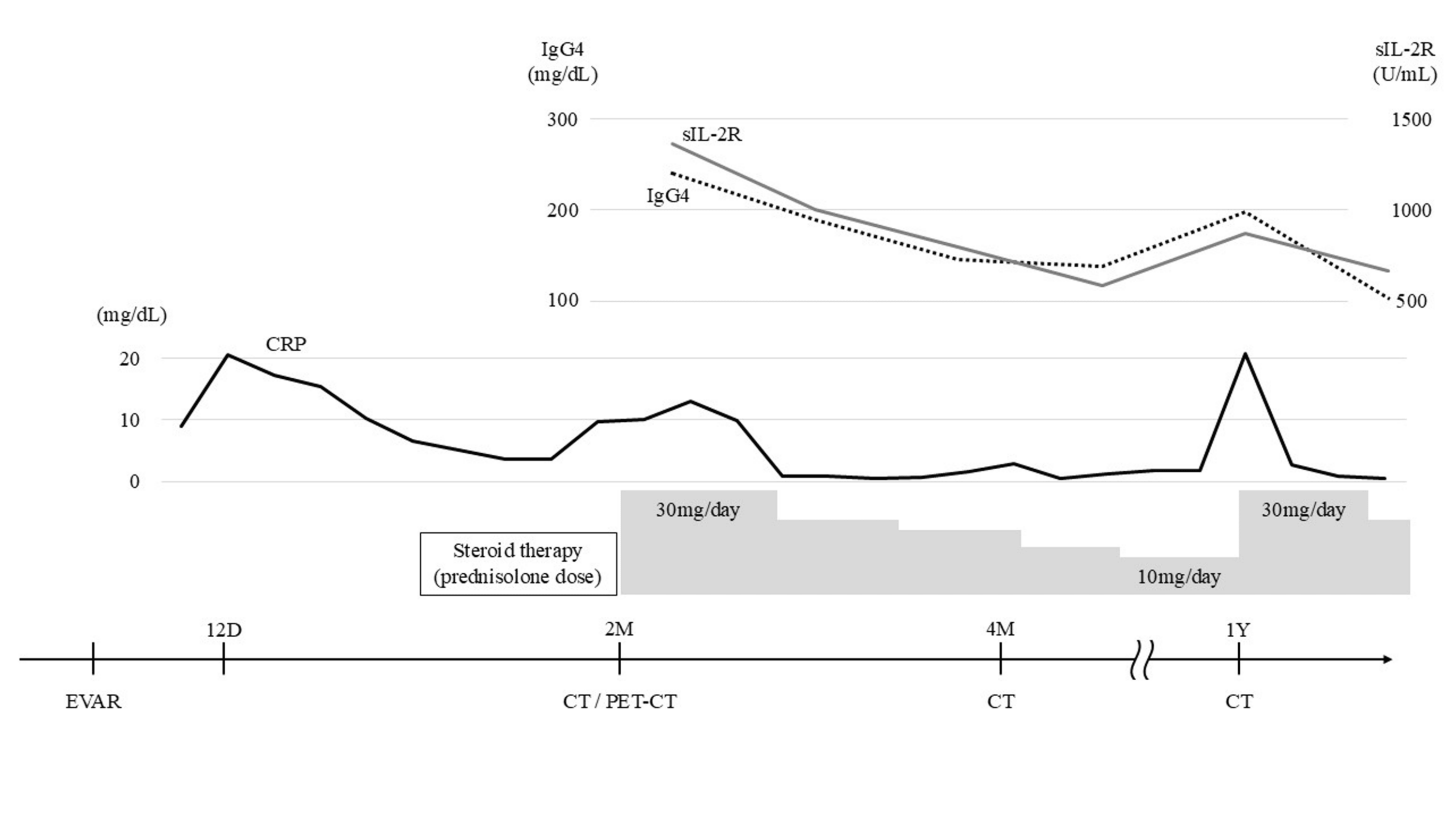
The course from EVAR to one year postoperatively The serum levels of IgG4 are shown as a dashed line, sIL-2R as a gray line, and CRP as a solid line. The prednisolone dose was gradually reduced, but it was increased during IA relapse. IgG4: immunoglobulin G4; sIL-2R: soluble interleukin-2 receptor; CRP: C-reactive protein; IA: inflammatory aneurysm; EVAR: endovascular aneurysm repair; CT: computed tomography; PET-CT: positron emission tomography-CT; 12D: 12 days; 2M: 2 months; 4M: 4 months; 1Y: one year

The prednisolone dose was then gradually reduced to 20 mg/day. During the post-EVAR, steroid therapy led to a decrease in the serum levels of IgG4 and CRP (Figure 4). The patient was discharged on day 24 and was being followed up with steroid therapy as an outpatient.

## Discussion

IgG4-RD is considered the most common cause of inflammatory AAAs [3]. It is characterized by abundant infiltrating lymphocytes and IgG4-positive plasma cells in various organs affected by fibrosis or sclerosis [3]. The combination of arterial wall thickening and periarterial inflammation is termed IgG4-related periarteritis [4]. Moreover, the combination of IgG4-related periarteritis and aneurysms is termed IgG4-related IAs. Even in the absence of preoperative inflammatory findings, EVAR occasionally causes IAs after the implantation of the stent graft [7-11].

After comprehensive review of the available literature, we identified 16 reported cases of post-EVAR IA, including the present case [7-11]. In all these cases, the clinical symptoms, inflammatory findings, and imaging features, which were indicative of IAs, were absent before EVAR. Among all these previous cases of post-EVAR IA, postoperative IgG4 levels were reported only by Sumino [9]. Pathological specimens should be obtained for the diagnosis of IgG4-related IAs. However, owing to the widespread adoption of EVAR and its recommendation for IAs in guidelines [2], a definitive diagnosis of IgG4-RD is difficult to obtain. In this case, we relied on CT characteristics and laboratory test results indicative of inflammation to diagnose IA. In this case, after confirming that various cultures were negative, oral prednisolone was administered as steroid therapy at a dose of 0.5 mg/kg/day for suspected IgG4-RD. In most previous reports of post-EVAR IA, steroid therapy was performed, the initial treatment was successful, and no relapses were reported [7-11]. In this case, during steroid reduction, inflammation recurred. The recurrent inflammatory findings demonstrated renewed improvement with an increase in the steroid dosage. Such a course of recurrence and remission is a characteristic of IgG4-RD [4], and the serum levels of IgG4 and sIL-2R were particularly helpful in assessing disease activity [12]. Zhang and Stone reported that risk factors for relapse include elevated serum IgG4 levels, a high IgG4-RD Responder Index (RI) score, and increased levels of serum immunoglobulin E or eosinophils at baseline [13]. The IgG4-RD RI, developed by Carruthers et al., is a standardized clinical tool used to evaluate disease activity in patients with IgG4-RD. It assigns a score from 0 to 4 to each affected organ system based on clinical severity and an additional score from 0 to 4 based on the serum IgG4 concentration. The total RI score is the sum of these values, with higher scores indicating greater disease activity [14]. In this case, the relapse of the IA occurred during the tapering of steroid therapy. The RI score was 9 both at baseline and at the time of relapse, and it decreased to 2 following an increase in steroid dosage. These findings are consistent with previous reports that relapse is common after glucocorticoid tapering, particularly in patients with high RI scores and elevated serum IgG4 levels [13]. A systemic inflammatory response may occur after EVAR in a proportion of patients and is referred to as PIS [15]. PIS is characterized by constitutional symptoms such as fatigue or other flu-like manifestations, fever, and laboratory findings of inflammation (e.g., elevated CRP or WBC count). Although PIS usually resolves spontaneously within a month without specific treatment, the present case showed persistent inflammation and radiological abnormalities, which are more consistent with an IgG4-related IA. The clinical course and response to steroid therapy support this diagnosis. However, as PIS typically resolves spontaneously within a month and without relapse, this case was diagnosed as IA with elevated IgG4 levels rather than a simple case of PIS (Figure 4). Reports have suggested that preoperative steroid administration is effective to prevent PIS, but it did not influence the clinical course following EVAR in this case [15]. While the latest guidelines mention PIS, they do not recommend preoperative steroid administration [2]; therefore, we did not do so in this case.

In this case, a definitive diagnosis of IgG4-related IA could not be made owing to the lack of pathological specimens. Another possibility is that the implantation of the stent graft triggered the inflammation. In fact, retroperitoneal fibrosis (RPF) is classified as IgG4-related if it meets certain pathological criteria [16]. RPF, also referred to as chronic periaortitis, is a fibroinflammatory disorder that encompasses a spectrum of entities, including idiopathic RPF, inflammatory AAAs, perianeurysmal fibrosis, and IgG4-related periaortitis. Given this overlap, distinguishing RPF from IgG4-related IA based on imaging or clinical features alone is often challenging [17]. Furthermore, current Japanese diagnostic criteria apply the same standards to both IgG4-related periaortitis and RPF, without providing specific imaging-based distinctions. Therefore, in the present case, we diagnosed the condition as an IA with elevated IgG4 levels following EVAR, acknowledging the difficulty in establishing a definitive subtype.

Based on the clinical course and findings, the patient in this case was diagnosed with IA with elevated IgG4 levels following EVAR. However, in the era of EVAR, diagnosing a case as IgG4-related remains challenging. Further accumulation of cases is awaited, and this topic warrants continued discussion.

## Conclusions

Here, we report a case of an IA with the elevation of serum IgG4 levels that was diagnosed after EVAR. Even in the absence of preoperative inflammatory findings, inflammation may become evident postoperatively. Steroid therapy appears to be an effective treatment option for post-EVAR IAs. The IgG4-RD RI, which combines organ involvement and serum IgG4 levels, was useful in assessing disease activity and monitoring relapse in this case. Considering the potential for recurrent inflammation, long-term follow-up is desirable for the management of patients with post-EVAR IAs.
